# Anthocyanins in Colorectal Cancer Prevention Review

**DOI:** 10.3390/antiox10101600

**Published:** 2021-10-12

**Authors:** Ni Shi, Xiaoxin Chen, Tong Chen

**Affiliations:** 1 Department of Internal Medicine, Division of Medical Oncology, The Ohio State University, 1800 Cannon Drive, 13th Floor, Columbus, OH 43210, USA; ni.shi@osumc.edu; 2 Comprehensive Cancer Center, The Ohio State University, Columbus, OH 43210, USA; 3 Cancer Research Program, Julius L. Chambers Biomedical Biotechnology Research Institute, North Carolina Central University, 700 George St., Durham, NC 27707, USA; lchen@nccu.edu

**Keywords:** colorectal cancer, anthocyanins, phytochemicals, berries, grapes, antitumor effect

## Abstract

Colorectal cancer (CRC) is still a big health burden worldwide. Nutrition and dietary factors are known to affect colorectal cancer development and prognosis. The protective roles of diets rich in fruits and vegetables have been previously reported to contain high levels of cancer-fighting phytochemicals. Anthocyanins are the most abundant flavonoid compounds that are responsible for the bright colors of most blue, purple, and red fruits and vegetables, and have been shown to contribute to the protective effects of fruits and vegetables against cancer and other chronic diseases. Berries and grapes are the most common anthocyanin-rich fruits with antitumor effects. The antitumor effects of anthocyanins are determined by their structures and bioavailability as well as how they are metabolized. In this review, we aimed to discuss the preventive as well as therapeutic potentials of anthocyanins in CRC. We summarized the antitumor effects of anthocyanins and the mechanisms of action. We also discussed the potential pharmaceutical application of anthocyanins in practice.

## 1. Introduction

Colorectal cancer (CRC) is the third most common tumor and the second leading lethal tumor worldwide [1]. The risk factors for CRC include heritable genetic changes, environmental and food-borne mutagens, excess weight and obesity, inflammatory bowel disease, heavy alcohol use, smoking, physical inactivity, compromised gut microbiota, and poor diet. Nutrition and dietary factors are known to affect CRC. Epidemiological investigations have revealed that diets high in fruits, vegetables, and whole grains are associated with reduced risk of CRC, while diets high in red and processed meat are associated with increased risk [2]. The American Cancer Society recommends limiting the intake of red and processed meats and eating more vegetables and fruits to help lower the risk of CRC. The protective roles of diets rich in fruits and vegetables have been previously reported. Fruit and vegetable-rich diets are associated with reduced oxidation, DNA damage, cell growth inhibition, cell cycle arrest, and low-grade inflammation as well as the promotion of beneficial gut microbiota [3]. Phytochemicals, naturally occurring plant chemicals that provide fruits and vegetables with color, odor, and flavor, show the potential to reduce oxidative stress, prevent DNA damage, impact DNA repair, slow cancer cell growth, promote cancer cell death, reduce inflammation, and boost immune function [4]. The most popular types of phytochemicals reported exhibiting anticancer efficacy contain carotenoids, flavonoids, isoflavones, polyphenols, and isothiocyanates. Anthocyanins are the most abundant flavonoid compounds that are responsible for the colors of most fruits and vegetables, and they contribute to the protective effects of fruits and vegetables against chronic diseases and cancer [5].

**Figure 1:**
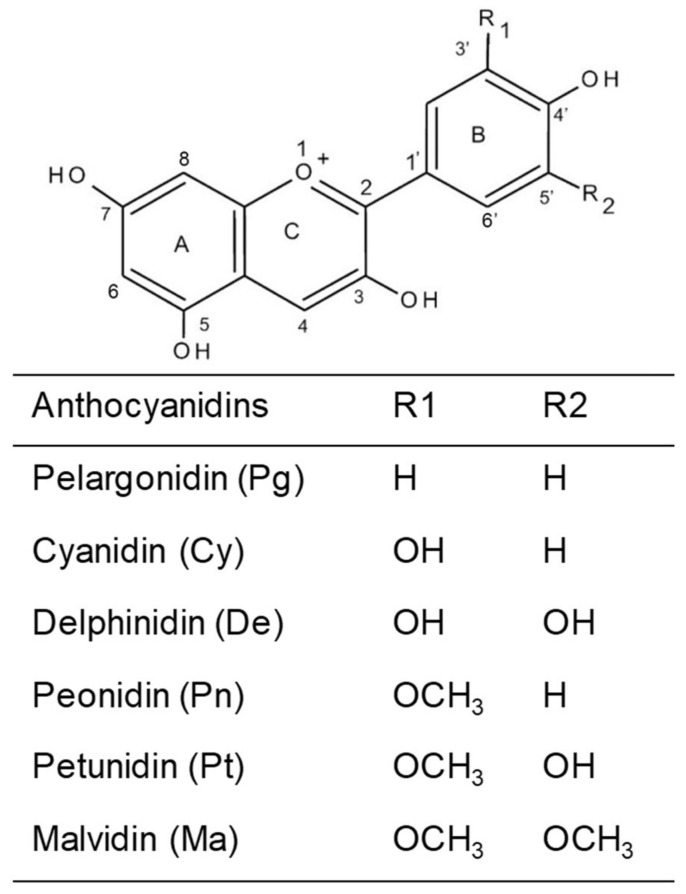
The structural classification of the six most common anthocyanins.

Anthocyanins are derived from flavonol with a basic structure of a flavylium ion lacking a ketone oxygen at the 4-position [6,7]. Depending on their skeleton, there are six different types of anthocyanidins: cyanidin, delphinidin, pelargonidin, peonidin, petunidin, and malvidin (Figure 1) [5]. Cyanidin is the major magenta pigment in berries and some red-colored vegetables such as purple corn, purple sweet potato, and red sweet potato. Delphinidin is a blue-reddish or purple pigment in the plant. Pelargonidin is the red-colored pigment in some fruits and berries. Peonidin, malvidin, and petunidin are three methylated anthocyanidins. Peonidin exists abundantly in berries, grapes, and red wines. Malvidin, the purple pigment, is the major dark-red pigment in natured red wine. Petunidin is a dark red or purple water-soluble pigment detected in black currants and purple flower petals. Anthocyanins carry different glucosides as the glycosylated form of anthocyanidin. Usually, the glucoside group of anthocyanins conjugate to the anthocyanidin skeleton via the C3 hydroxyl group in ring C. The most commonly found glucosides that bond to anthocyanidins have glucose (glc), galactose (gal), arabinose (ara), rutinose (rut), rhamnose (rham), and xylose (xyl) in mono-, di-, or trisaccharide forms [8].

The chemical structure of anthocyanins is significantly associated with their stability and bioavailability, both of which determine their health benefits. Most anthocyanins are not stable and are sensitive to light, temperature, pH, enzymes, oxygen, antioxidants, and other factors, necessitating high standards for the anthocyanin regimen and supplement product.

The bioavailability and metabolism of anthocyanins are of great research interest in biomedicine, clinic, and pharmacology. The rate and extent of anthocyanin absorption are affected by the anthocyanidin, sugar moiety, and acylated groups in the chemical structure. Anthocyanins can be absorbed via the mouth, stomach, and small intestine. Anthocyanins can be taken up, hydrolyzed, and conjugated by oral epithelial cells, and oral bacteria [9]. Anthocyanins can permeate the gastric mucosa through a bilitranslocase-mediated mechanism [10]. Anthocyanin glycosides can be rapidly and efficiently absorbed in the small intestine, involving transportation mechanisms such as glucose transporter SGLT1, glucose-associated transporters (GLUTs), and intestinal bilitranslocase [10,11,12,13]. In the colon, the absorption of anthocyanins is zero, but the metabolism of anthocyanins will occur due to spontaneous degradation under physiological conditions or microbial metabolism [11,14]. However, only a small amount of anthocyanins can be found in the circulatory system and in the urine. The gap between the poor bioavailability and the positive health effects of anthocyanins is still a big challenge for the pharmacology research and development of anthocyanin-derived drugs. Extensive first-pass metabolism of anthocyanins is one of the reasons for the poor bioavailability, which means that most anthocyanins enter the circulatory system as metabolites [8,11,12,15]. The total bioavailability of anthocyanins should be higher than the estimated approximate 1% in previous reports when accounting for unmetabolized parent compounds, phase I and phase II metabolites, conjugated products, and microbe-generated metabolites [8,11,12,15]. However, the complex mixtures of unmetabolized parent anthocyanins, phenolic degradation products, glucuronide conjugates, sulfides conjugates, and post-colonic bacterial metabolites may coexist in the biofluids and tissues. Which anthocyanins are subject to the specific metabolism is not well known, and the technology to detect all the metabolites is also limited. Scientific understanding of the bioavailability and metabolism of anthocyanins is constantly evolving.

In our former studies, we demonstrated the cancer preventive effects of strawberries and black raspberries in colon cancer and esophageal cancer and found that anthocyanins are the main components in both fruits [16,17]. The roles of strawberry and black raspberry (BRB) extracts and anthocyanin compounds in colon cancer cell lines or animal models are summarized in our latest review on strawberries and BRB in inflammation-related CRC prevention [5]. Anthocyanins exist abundantly in fruits, vegetables, legumes, nuts, tea, and wine. Aside from strawberries and BRB, other fruits and vegetables rich in anthocyanins such as cherry, grape, pomegranate, purple sweet potato, eggplant, and some dark leafy greens also showed anticancer effects against CRC in both in vitro and in vivo models [16,17]. In this review, we summarize the anticancer effects of anthocyanins in CRC, and then discuss the pharmaceutical application of anthocyanins in CRC prevention and therapy.

## 2. Anthocyanins in Colon Cancer Prevention

### 2.1. Epidemiology Study

Some epidemiology studies have found an inverse association between the high consumption of anthocyanins and the decreased risk of CRC. A large multicentric case-control study conducted in Italy found a significant inverse relationship between anthocyanidins and CRC, with a hazard ratio of 0.78 (95% CI, 0.61–0.99) adjusting age, sex, fruit and vegetable intake, BMI, physical activity, alcohol intake, and education level [18]. In another case-control study in China, no significant association was found between the total flavonoid intake and CRC risk, but an inverse association between anthocyanidins and CRC risk was verified with an adjusted OR of 0.80 (95% CI 0.64, 1.00) for the highest quartile for anthocyanidin consumption compared with the lowest quartile [19]. However, other studies have shown no association between anthocyanin intake and CRC risk [20,21,22,23]. A more recent meta-analysis indicates that anthocyanin consumption is inversely associated with the risk of developing CRC with a pooled RR at 0.78 (95% CI, 0.64–0.95) [24]. Regarding the critical roles of systematic review and meta-analysis in evidence-based medicine, this finding significantly supports the active role of anthocyanins in CRC prevention.

### 2.2. Anthocyanin-Rich Whole Fruits and Vegetables in Colon Cancer Prevention

In our previous study, we detected the role of lyophilized strawberries in azoxymethane (AOM)/dextran sodium sulfate (DSS)-induced and inflammation-associated colon carcinogenesis in the murine model. We found that strawberries decreased tumor incidence, reduced a bundle of proinflammatory mediators, and the nitrosative stress, at least, through inhibiting phosphatidylinositol 3-kinase (PI3K)/AKT and nuclear factor kappa B (NFkB) signaling cascades, as indicated in Table 1 [16]. Other groups also found that lyophilized blackberries and strawberries reduce the tumor multiplicity and pro-inflammatory gut bacteria in the AOM/DSS murine model [25]. In our recently published review, we summarized the roles of strawberries, BRB, and anthocyanin components in the chemoprevention of inflammatory bowel disease (IBD)-related CRC [5]. In the rat model of colitis-associated carcinogenesis induced by 1,2-dimethylhydrazine (DMH) and 2,4,6-trinitrobenzene acid (TNBS), lyophilized acai pulp can reduce aberrant crypt foci (ACF) multiplicity, tumor cell proliferation, and incidence of tumors with high-grade dysplasia [26]. IBD patients are at an increased risk of developing CRC. CRC that develops in IBD patients tends to have a worse prognosis and survival rate compared to patients with sporadic CRC in the advanced stage. Compared to the modest efficiency of some chemical inhibitors, the anthocyanin-rich whole fruits showed better efficiency in preventing CRC in IBD patients due to additive and synergistic activities of multiple bioactive phytochemicals [5].

For sporadic CRC, BRB showed preventive effects in the non-inflammation-promoted CRC model, the AOM rat model. BRB decreased the ACF burden, ACF, and adenocarcinoma multiplicities, and reduced urinary 8-hydroxy-2′-deoxyguanosine (8-OHdG) levels [27]. Pomegranate and baked purple potato were also shown to prevent CRC development in the AOM-induced CRC in animals. Pomegranate juice suppressed the number of ACF and dysplastic ACF, lowered proliferation of mucosa cells and decreased expression levels of cyclooxygenase-2 (COX-2), inducible nitric oxide synthase (iNOS), NF-κB (p65), and vascular cell adhesion molecule 1 (VCAM-1) [28]. Baked purple potato suppressed the tumor incidence, promoted apoptosis, and increased cancer stem cell markers [29]. Apc^Min^ mouse is a popular animal model for studies of human colon cancer. With a mutation in the APC gene, the primary phenotype of Apc^Min^ mice is the development of multiple intestinal adenomas [30]. Tart cherry significantly decreased the number and volume of adenomas in Apc^Min^ mice [31]. As in IBD-related CRC, anthocyanin-rich whole fruits and vegetables are efficient strategies to prevent sporadic CRC.

### 2.3. Anthocyanin Extract in Colon Cancer Prevention

The pros for whole foods in animal studies are the additive and synergistic activities of multiple bioactive phytochemical profiles. These pros are also cons for the whole food study, as it is unclear as to which components are responsible for chemoprevention. We considered anthocyanins to be largely responsible for the chemoprevention effects of anthocyanin-rich fruits and vegetables. The anthocyanin extract from these foods showed significant efficiency in preventing colon cancer in cell lines, animal models, and clinical trials (Table 2).

#### 2.3.1. Black Raspberry

In CRC cell lines, the anthocyanin-enriched extract of BRB can suppress cell proliferation, induce apoptosis, and decrease the activity of DNMT1 and DNMT3B, and demethylate CDKN2A, SFRP2, SFRP5, and WIF1 in the Wnt pathway [32,33]. In the AOM/DSS colon cancer mouse model, the BRB anthocyanin extraction in the diet decreased tumor multiplicity, modulated the composition of gut commensal microbiota, and changed the inflammation index and the methylation status of the SFRP2 gene [34].

#### 2.3.2. Blueberry

Blueberry extract inhibited the cell proliferation of Caco-2 cells with an IC_50_ at 0.78 ± 0.15 mM [35,36]. In addition, blueberry extract inhibited cell proliferation and promoted apoptosis in HCT116 and HT-29 cells [33,36]. Compared to phenolic acids, tannins, and flavonol components, the blueberry anthocyanin extract showed the greatest antiproliferation effects. The anthocyanin extract from China blueberry (*Vaccinium uliginosum L*) inhibited proliferation in DLD-1 and COLO205 cells [37].

#### 2.3.3. Bilberry

The bilberry anthocyanin extract showed antioxidative activity in Caco-2 cells, with an IC_50_ at 0.53 ± 0.04 mM [35]. Bilberry showed an antiproliferation effect in HT-19 cells and the mechanisms associated with increasing the expression of p21 [38,39,40]. Bilberry anthocyanin extract induced mitochondrial damage, activated apoptosis, inhibited cell proliferation in the mouse colon cancer cell line MC38 [41]. Bilberry extract has the potential to reduce the side effects of some chemotherapy drugs by suppressing the activity of topoisomerase I and the DNA strand breaks [42]. In the AOM/DSS-induced and IBD-related CRC mouse model, the bilberry anthocyanin extract decreased the tumor number and inflammation [43]. In the AOM rat model, the bilberry extract was shown to reduce total ACF, colonic cellular proliferation, and COX-2 mRNA expression [44].

#### 2.3.4. Cranberry

The cranberry anthocyanin extract inhibited the cell proliferation of HT-29, SW620, and SW480 cells derived from the primary tumor and the metastatic site of the same patient with CRC [33,45]. Interestingly, the cranberry anthocyanin extract exhibited more potent inhibitory effects in the progressive and metastatic SW620 cells, but not SW480 cells [45].

#### 2.3.5. Chokeberry

The chokeberry anthocyanin extract inhibited the growth of HT-29 cells [38,40], and reduced the total number of ACF and colonic cellular proliferation in the AOM rat model [44]. A special black chokeberry (*Aronia meloncarpa E*) exhibited antioxidative effects, inhibited cell growth, promoted cell phase arrest, and upregulated several cell cycle-related genes [46,47,48,49].

#### 2.3.6. Blackberry

The blackberry anthocyanin extract showed antioxidant, antiproliferative, and anti-inflammatory activities in HT-29 and Caco-2 cells by inhibiting cell growth and interleukin (IL)-12 release from mouse bone marrow-derived dendritic cells [33,50,51].

#### 2.3.7. Other Berries

Other berries like red raspberry and strawberry extract, lingonberry, elderberry, and black currant showed antitumor effects in CRC cell lines by inhibiting cell growth and promoting cell apoptosis [33,52]. The anthocyanin extract from flowering plant berries such as *Prunus spinosa drupes* (blackthorn) could inhibit the growth and colony formation, promote apoptosis in HCT-116 cells, and reduce tumor growth in xenograft mice [53].

#### 2.3.8. Grapes

Besides berries, grapes are of great interest for research due to their rich anthocyanin profiles and noted health benefits for humans. In HT-29 and CaCo-2 cells, the anthocyanin-rich extracts from grapes could inhibit cell growth and promote apoptosis [38,54,55]. The grape extract has the potential to reduce the side effects of some chemotherapy drugs by suppressing the activity of topoisomerase I and the DNA strand breaks [42]. Compared to phenolic acids, tannins, and flavonol components, the grape anthocyanin extract showed the greatest antiproliferation effects. In another study, however, the authors found that the non-anthocyanin fraction showed a better antitumor effect [56]. In the AOM rat model, the grape extract reduced the total burden of ACF and COX-2 mRNA expression [44]. In APC^min^ mice, the grape pomace extract decreased adenoma burden, adenoma number, and the expression of AKT and Ki-67 [57].

#### 2.3.9. Red Wine

Red wine contains anthocyanins as well as flavonoids and their derivatives and shows various promising biological effects. The methanol extracts of red wine could suppress HCT-15 cell growth and block the S, G2, and M phases [58]. The anthocyanin fraction showed higher efficiency than the non-anthocyanin fractions. In HCT-116 cells, the lyophilized red wine extract reduced cell proliferation, increased the expression of p53, p21 cell cycle gatekeepers, induced antioxidant response by activating transcriptional factor, nuclear factor erythroid 2–related factor 2 (Nrf2), and promoted differentiation through E-cadherin and the epithelial-mesenchymal transition (EMT) pathway [59].

#### 2.3.10. Tart Cherry

Similar to whole tart cherry, anthocyanin-rich extract from tart cherry could decrease bodyweight loss and tumor burden when combined with sulindac in APC^min^ mice [60].

#### 2.3.11. Plum

Polyphenol-rich extract from Illawarra plum (*Podocarpus elatus Endl.*) suppressed cell growth, arrested S cell phase, increased apoptosis, and decreased telomerase activity and telomere length in HT-29 cells [61]. The anthocyanin extract from Java plum (*Eugenia jambolana*) suppressed cell proliferation, promoted apoptosis in HCT-116 cells, and elevated the proportion of colon cancer stem cells [62].

#### 2.3.12. *Vitis coignetiae Pulliat*

Grape family fruit *Vitis coignetiae Pulliat* (*Meoru* in Korean) has been used in Korean folk medicine to treat inflammatory disorders and cancer for a long time. The intense dark red hue of *Vitis coignetiae Pulliat* indicates an abundance of anthocyanin pigments [63]. Anthocyanins isolated from Meoru inhibited cell viability, proliferation, motility, and invasiveness, and induced cell apoptosis in HCT-116 and HT-29 cells [63,64,65,66]. In the HT-29 xenograft mouse model, the anthocyanins isolated from Meoru significantly suppressed tumor growth [65].

#### 2.3.13. Purple Fleshed Sweet Potato

Purple fleshed sweet potato is a specific sweet potato rich in anthocyanins [67]. The anthocyanin extract from purple fleshed sweet potato decreased cell number, inhibited cell growth, and promoted cell cycle arrest in WiDr and SW480 cells [67,68]. In colon cancer stem cells, anthocyanin extraction suppressed proliferation, promoted apoptosis, and suppressed the WNT signaling pathway [29]. In the mouse model of AOM-induced CRC, anthocyanin extraction decreased the ACF number, cell proliferation, and promoted apoptosis [67].

#### 2.3.14. Other Fruits and Vegetables

The anthocyanin extract from purple-shoot tea [69], curly kale [70], Chinese eggplant [71], and *Pistacia atlantica sub kurdica* [72], and apple [73] showed antitumor effects in cell lines or carcinogenesis animal models. The antitumor effects are associated with cell proliferation inhibition, apoptosis induction, cell migration inhibition, cell cycle arrest, DNA damage protection, and antioxidation.

### 2.4. Anthocyanin Single Compounds and Their Metabolites in Colon Cancer Prevention

#### 2.4.1. Cyanidin

Cyanidin is one of the most widespread anthocyanidins in fruits and vegetables. Aglycone cyanidin can inhibit CRC cell growth and proliferation [74,75,76]. In Cvorovic’s study, they found that the cell toxicity was higher in drug-resistant cancer cells than that in their parental cells [76]. In Briviba’s study, the author found that aglycone cyanidin showed better effects than its glycosides, cyanidin chloride-3-5-diglucoside and cyanidin chloride-3-galactoside [75]. In the study by Renis, they reported that cyanidin-3-*O*-β-glucopyranoside can inhibit cancer cell growth and proliferation with different molecular effects than the aglycone cyanidin [74].

The purified cyanidin-3-glucoside from strawberries showed antioxidant effects as well as the ability to inhibit the cell growth of HT-29 and HT-116 cells. Moreover, cyanidin-3-glucoside had the best antioxidant effects among the anthocyanins and other phenolics extracted from strawberries [77]. The cyanidin-3-glucoside single component promoted colon cancer cell attachment to fibronectin and inhibited 3D spheroid growth [78]. Cyanidin-3-*O*-β-glucoside extracted from purple corn can decrease the development of colonic mucosal lesions and ACF induction in DMH-PhIP-treated animals [79]. Cyanidin 3-cyanidin 2′′-*O*-β-D-glucopyranosyl-6′′-*O*-α-rhamnopyransyl-β-D—glucopyranoside and 3-cyanidin 6′′-*O*-α-L-rhamnopyranosyl-β-D-glucopyranoside isolated from tart cherry decreased the number and volume of adenomas in APC^min^ mice, and reduced the cell growth of the HT-29 and HCT-116 cells [31].

#### 2.4.2. Delphinidin

Compared to cyanidin, delphinidin differs in the number and position of hydroxyl groups on the b ring in the molecular structure. Delphinidin inhibited cell growth, decreased cell viability, arrested the G1 and G2/M cell phases, and promoted apoptosis in the Caco-1 and HCT-116 cells [80,81,82]. The cell toxicity of delphinidin is higher in drug-resistant cancer cells than that in their mother cells [76].

#### 2.4.3. Pelargonidin

Pelargonidin, pelargonidin-3-glucoside, and pelargonidin-3-rutinoside isolated from strawberries can inhibit the cell growth of HT-29 and HCT-116 cells [77]. Pelargonidin, but not pelargonidin-3-glucoside or pelargonidin-3-rutinoside, has the best antioxidant efficiency.

#### 2.4.4. Metabolites

Gut anthocyanin metabolites, gallic acid, 3-*O*-methylgallic acid, and 2,4,6 –trihydroxybenzaldehyde reduce cell proliferation more effectively than anthocyanins in Caco-2 cells [83]. In addition, gallic acid and 3-*O*-methylgallic acid decreased cell viability, arrested G0/G1 cell cycle, promoted apoptosis, and caused DNA fragmentation and nuclear condensation [84,85]. The effects of single anthocyanin components and their metabolites in cancer prevention of CRC are summarized in Table 3.

## 3. Molecular Mechanisms Associated with Chemopreventative Effects of Anthocyanins in Colon Cancer

### 3.1. Antioxidant

The most well-known mechanism associated with the health-protective effects of anthocyanins is antioxidant activity. Oxidative stress is a complex condition involving reactive oxygen species (ROS) (ONOO^−^, O^2−^, H_2_O_2_, and LPO) and antioxidants (GPx and SOD_2_). Oxidative stress occurs as a response to increased ROS or decreased antioxidants, leading to a failure to prevent ROS-induced oxidative damage [86]. Anthocyanins are reported to have superior antioxidant properties compared with other related polyphenols and well-recognized antioxidants such as ascorbic acid and α-tocopherol [77,87]. Anthocyanins were reported to suppress oxidative damage, DNA strand-breaking effects, transepithelial electrical resistance in colon cancer cells, and decreased oxidative stress marker urinary 8-OHdG levels in animal models of AOM-induced CRC [27,35,42,47,51,63,71,76,81,86,88].

Anthocyanins are antioxidative by scavenging ROS, promoting glutathione reductase expression involved in metal chelation, inhibiting lipoprotein oxidation, inhibiting peroxyl radical-induced apoptosis, and protecting the formation of oxidative DNA damage [47,51,71,76,86,88]. The antioxidant capacity of anthocyanins is affected by their structures. Hydroxyl groups in position 3 of ring C and the 3′, 4′ and 5′ positions in ring B are the major components responsible for the antioxidant activity. Generally, cyanidin with groups 3′, 4′ di-OH substituted has higher antioxidant activity when compared to pelargonidin, malvidin, and peonidin with a single OH group in ring B [89]. However, delphinidin still has low antioxidant activity even with groups 3′ and 4′ di-OH substituted. Types of glycosylation of anthocyanidins are also important for their antioxidant activities. Different glycosylation patterns either enhanced or reduced the antioxidant capacity [71]. In addition, the antioxidant capacity of anthocyanins is impacted by the food intake of anthocyanins.

The critical molecules involved in antioxidation include the PI3K/AKT signaling pathway, NFκB/mitogen-activated protein kinase (MAPK) signaling pathway, Nrf2, p27, HSP70, and 8-oxoguanine DNA glycosylase-1 (OGG1) [59,74,86,90]. The molecular mechanisms associated with antioxidation differ among anthocyanins. For example, the decreased ROS caused by cyanidin chloride is due to the upregulation of HSP70. Cyanidin-3-*O*-β-glucopyranoside not only dose-dependently modulated ROS level, but also decreased DNA fragmentation by non-dose-dependently regulating HSP70 [74]. However, anthocyanins might prevent colon carcinogenesis through multiple biological processes aside from antioxidation.

### 3.2. Antiproliferation

Anthocyanin-rich whole foods, anthocyanin extracts, anthocyanin single compounds, and anthocyanin metabolites have all exhibited antiproliferation activities in multiple colon cancer cell types and animal models. In parallel with the antiproliferation effects, anthocyanins arrested the cell cycle at G1/G0 and G2/M, and modulated cell cycle-related genes (cyclin A, cyclin B, cyclin E, cyclinD1, p21, p25, p53, and cyclin-dependent kinase inhibitors) [46,58,61,80,81]. The MAPK pathway (ERK, c-Jun-NH2 kinase, and p38) represents a superfamily of proteins that regulate cell differentiation, proliferation, and survival during carcinogenesis [16]. Blocking activation of the MAPK pathway was reported as one of the molecular mechanisms for the anticarcinogenic action of anthocyanidins in colon cancer [16,63,64,91]. Inhibition of the receptor tyrosine kinases (RTKs) such as epidermal growth factor receptor (EGFR), ErbB2, ErbB3, vascular endothelial growth factor receptor-2 (VEGFR-2), VEGFR-3, and insulin-like growth factor 1 receptor (IGF1R) might be another molecular event associated with the antiproliferation effects of anthocyanins in cancers [92,93]. Cyanidin-3-Oglucoside and delphinidin-3-*O*-glucoside significantly inhibited EGFR in HCT-116 and HT-29 cells [55]. AKT/ERK/NFκB signaling cascades play an important role in cell proliferation and survival [5]. Lyophilized acai pulp increased the negative regulators of cell proliferation such as Dlc1 and AKT3 [26]. Pomegranate juice inhibited the phosphorylation of PI3K/AKT and mTOR expression and suppressed NFκB in the AOM rat model [28]. Lyophilized strawberries decreased PI3K/AKT signaling and NFkB in the AOM/DSS mouse model [16]. Grape anthocyanin extract from red grape (*Vitis coignetiae Pulliat*) has inhibitory effects on PI3K/AKT and mTOR [57,63,65]. A decrease in both hTERT expression and telomere length was reported as one of the mechanisms of the antitumor effects of Illawarra plum in colon cancer [61].

The antiproliferation effects of anthocyanins are also affected by their structures. The ortho-dihydroxyphenyl structure on the B-ring was considered as the functional group contributing to the inhibitory action [94]. The nonacylated monoglycosylated anthocyanins have higher effects than anthocyanins with pelargonidin, triglycoside, and/or acylation with cinnamic acid exerted [40,71]. For each anthocyanin, the exact mechanisms for these potential anticancer activities differ from one another.

### 3.3. Induction of Apoptosis

Apoptosis is another key target for antitumor activities. Anthocyanin-rich whole foods, anthocyanin extract, anthocyanin single compounds and anthocyanin metabolites have all exhibited apoptosis-inducing activities in multiple colon cancer cell types and animal models. The pro-apoptotic effect of anthocyanins can be attributed to the ROS-mediated mitochondrial caspase-independent pathway [62,82,88]. Anthocyanin extract from purple-shoot tea promoted apoptosis through cleavage of poly adenosine diphosphate-ribose polymerase (PARP), activation of caspase 3, and an increased expression of the Bax/Bcl-2 ratio [69]. Anthocyanin extracts and single compounds promoted apoptosis by decreasing the expression of anti-apoptotic proteins (survivin, cIAP-2, and XIAP) [55]. Anthocyanins could promote apoptosis in a p53-independent manner in colon cancer stem cells by elevating Bax and cytochrome c, proteins-mediating mitochondrial apoptosis [29,82].

### 3.4. Anti-Invasive Activity

Cancer cells can spread beyond the layer of tissue where the initial tumor developed and grow into nearby healthy tissues. Anthocyanin extract from *Vitis coignetiae Pulliat* inhibited HT-29 cell invasion by suppressing the PI3K/AKT pathway and NFkB-MMP2/MMP-9 axis [64,66]. The EMT broadly regulates cancer cell invasion and metastasis. Anthocyanin extract from grapes can induce morphological change, increase the epithelial marker E-cadherin, and reduce the cell migration activity in HCT-116 cells [59]. Cancer stem cells (CSCs) are a subpopulation of cancer cells with unique properties of self-renewal, infinite division, and multi-directional differentiation potential [95]. CSCs play critical roles in colon cancer invasion and metastasis. Anthocyanin extract from grapes can significantly decrease the expression of colon CSCs markers CD44 and CD133 [59]. Anthocyanins extracted from baked purple-fleshed potato and Eugenia jambolana can inhibit proliferation and promote apoptosis in both colon cancer cells and colon CSCs [29,62]. The suppression of β-catenin, the WNT pathway effector and the key regulator of CSCs proliferation, contributed to the inhibition of CSCs by anthocyanins. β-catenin and E-cadherin regulation were also found in CRC patients consuming BRB powder [96].

### 3.5. Gene Demethylation

BRB anthocyanins decreased the expression of DNA methyltransferase and demethylated the hypermethylated promoters of the secreted frizzled-related protein 2 (SFRP2) gene to induce colon cancer carcinogenesis in the AOM-DSS mouse model [5]. In CRC patients, dietary BRB decreased the methylation of tumor suppressor genes, SFRP2, paired box 6a (PAX6a), Wnt inhibitory factor 1 (WIF1) [96]. In colon cancer cell lines, anthocyanin extract from BRB suppressed the activity and protein expression of DNA methyltransferase enzymes, DNMT1 and DNMT3B, and demethylated WNT upstream regulators, CDKN2A, SFRP2, SFRP5, and WIF1 [32].

### 3.6. Anti-Inflammation

Chronic inflammation induces oncogenic mutations, genomic instability, immune microenvironment changes, early tumor promotion, angiogenesis, and increased risk of CRC [5]. Reduction in inflammation by anti-inflammatory pharmaceuticals such as NSAIDs and 5-aminosalicylate showed chemopreventive effects for CRC. Anthocyanin-rich whole foods showed anti-inflammatory effects in multiple animal models.

Lyophilized strawberries reduced proinflammatory mediators, tumor necrosis factor-α (TNF-α), IL-1β, IL-6, and nitrosative stress [16]. Bilberry anthocyanin-rich extract decreased the colon length in AOM/DSS mice [43,44]. Cytokine GM-CSF and IL-8 were also found to be decreased by BRB powder in patients with CRC [32]. Anthocyanins were also shown to directly regulate cytokine and immune effectors. Blackberry extract suppressed lipid A-induced interleukin-12 released from mouse bone marrow-derived dendritic cells [50]. Single anthocyanins, cyanidin-3-rutinoside and quercetin-3-rutinoside, inhibited myeloid-derived suppressor cell (MDSC) expansion and modulated T lymphocyte proliferation [97]. NFκB regulates multiple genes that drive inflammatory responses including COX-2 and iNOS, which can promote the development of CRC [16]. Pomegranate juice decreased expression of COX-2, iNOS, NF-κB (p65), and VCAM-1 in AOM-treated rats [28]. Lyophilized strawberries inhibited the PI3K/AKT and NF-κB signaling pathway in tandem with a reduction in proinflammatory mediators and nitrosative stress [16]. Bilberry and grape anthocyanin extract can decrease mRNA expression of COX-2 in AOM-treated rats [44]. Delphinidin-3-*O*-glucoside, cyanidin-3-*O*-glucoside, delphinidin (DC) and gallic acid (GA) decreased the immune checkpoint, programmed cell death protein-1 (PD-1) and programmed death-ligand-1 (PD-L1), in HCT-116 and HT-29 cells, and thus showed the potential to activate the immune response in the tumor microenvironment and induce cancer cell death [98]. Although the anti-inflammatory effects of anthocyanins are broadly reported, the details on CRC prevention are limited. The role of anthocyanins in inflammation and immune effectors remains a challenging area of research.

### 3.7. Microbiota

Alterations in the composition and function of bacterial microbiota are considered significant factors for developing inflammation-promoted CRC [5]. The colon is the major place for anthocyanin metabolism due to microbial metabolism [11,14]. Anthocyanins showed both bacteriostatic and bactericidal activities in vitro [99]. Therefore, investigations on anthocyanins and microbiota play critical roles in elucidating the health benefits of anthocyanins as they relate to colon cancer. A cross-sectional study showed that the higher intake of anthocyanin-rich food was associated with higher microbial diversity [100]. Diets containing the anthocyanin extract from blackberries and strawberries significantly suppressed the populations of the pro-inflammatory *Bilophila wadsworthia* in AOM/DSS-treated animals [25]. Diets containing BRB modulate the imbalance in gut microbiota during carcinogenesis by decreasing the pathogenic bacteria *Desulfovibrio* sp. and *Enterococcus* spp., and increasing probiotics *Eubacterium rectale*, *Faecalibacterium prausnitzii*, and *Lactobacillus* in AOM/DSS-induced inflammation-associated CRC [34]. In the colitis mouse model, anthocyanins can reverse the DSS-induced reduction in *Porphyromonadaceae, Rikenellaceae Prevotellaceae, Firmicutes/Bacteroidetes* ratio, and decrease *Ruminococcus gnavus* [101,102]. The beneficial microbiota activities of anthocyanins are frequently reported in multiple disease animal models and human clinical trials. In C57BL/6 mice, blueberry anthocyanin extract increased *Bifidobacterium*, *Lactobacillus*, *Roseburia*, *Faecalibaculum*, and *Parabacteroides* [103]. In the mouse model, bileberry anthocyanins reduced the ratio of *Firmicutes/Bacteroidetes (F/B),* and increased *Akkermansia* and *Parabacteroides* [104]. In human clinical trials, anthocyanin-rich apple products increased *Sutterella*, *Butyricicoccus,* and *Lactobacillus* and reduced *Streptococcus*, *Ruminococcus*, *Blautia*, and *Roseburia* [105]. More research is needed to better understand how anthocyanins modulate the gut microbiota and thus regulate their bioavailabilities and antitumor activities.

## 4. Future Directions in Anthocyanin Application

### 4.1. Encapsulation

The low bioavailability of anthocyanins is a pitfall for clinical applications. Encapsulation of anthocyanins or their bioactive metabolites has the potential to increase the localized concentration and boost their health benefits such as chemoprevention for CRC. Anthocyanin nanocomplexes with chitosan hydrochloride (CHC), carboxymethyl chitosan (CMC), and beta-Lactoglobulin (beta-Lg) can significantly improve the stability and bioavailability of anthocyanins in a simulated gastrointestinal tract [106]. Oidtmann’s study also showed that encapsulation can inhibit the early degradation of anthocyanins in the intestinal system [107]. The biological properties, especially the antioxidant effects, of bilberry extract-loaded pectin amide core-shell capsules, whey protein matrix capsules, and coated apple pectin matrix capsules were comparable to those of the non-encapsulated bilberry extract [108]. In colon cancer cell line HT-29, anthocyanins encapsulated within the FA-g-MD wall showed higher antioxidant activities than anthocyanins alone [109]. Endogenous nanoparticle exosomes can be used to encapsulate anthocyanins in order to increase their availability and efficiency. In Aqil’s study, milk exosome-encapsulated anthocyanins had higher antiproliferation efficacy in ovarian cancer cell lines [110]. Moreover, encapsulation may have the possibility of regulating the gut microbial composition, and the biosynthesis of short-chain fatty acids by the gut microbiota [111].

### 4.2. Combination

The antitumor activity of anthocyanins can be affected by their structures. Methylation by catechol-*O*-methyltransferase (COMT) during metabolism will decrease the efficiency of anthocyanins [112]. The antiproliferative activity of anthocyanins extracted from grape anthocyanins, cyanidin-3-glucoside and delphinidin-3-glucoside, was improved when used in combination with the drug Entacapone, a COMT inhibitor [113]. The improved efficacy may be due to the higher bioavailability by inhibition of COMT and an increase in ROS.

To counteract their low bioavailability, anthocyanins can be combined with different anthocyanins, other polyphenols, and chemotherapy drugs [114,115]. Luteolin is a common plant flavonoid with the potential for cancer prevention and treatment. The combination of cyanidin-3-*O*-glucoside chloride and luteolin had higher efficiency of antiproliferation and apoptosis induction than either compound on its own [116]. The binding of pectin to anthocyanins can improve the stability of anthocyanins to enhance their colonic accessibility and gut microbial fermentation [117]. When combined with chemotherapy drugs, anthocyanins had the potential to decrease their side effects and improve efficacy. In the APC^min^ mouse model, anthocyanin-rich tart cherry extract combined with sulindac had less significant body weight loss, fewer tumors, and a smaller total tumor burden [60]. Delphinidin showed the activity of autophagy induction in hepatocellular carcinoma cells. When delphinidin was combined with an inhibitor of autophagy, 3-methyladenine, the macroautophagy was significantly magnified [118]. Petunidin-3-*O*-glucoside combined with a PI3K inhibitor significantly increased cell death in glioblastoma cells [119]. Delphinidin selectively sensitized leukemia cell NB4 to As(III) by decreasing intracellular arsenic accumulation and strengthening intrinsic/extrinsic pathway-mediated apoptosis induction [120,121].

### 4.3. Gut Microbiota Fermentation

Low bioavailability and complex metabolite profiles suggest that metabolites play an important role in the chemopreventive effects of anthocyanins. Gut microbiota is crucial in anthocyanin metabolism, as gut microbiota fermentation can help to formulate active metabolites of anthocyanins with antitumor activities. The metabolites produced by fermentation varies by the composition of microbiota and the chemical structure of anthocyanins [122]. New phenolic compounds such as syringic acid, rhamnetin, hippuric acid, cinammic acid, protocatechuic acid, caffeic acid, kaempferolrhamnoside, chlorogenic acid, cryptochlorogenic acid, ferulic acid, etc., are commonly found as the metabolites of anthoycanins after gut microbiota fermentation. Interestingly, some disparities in the metabolite profile of anthocyanins by different gut microbial strains were also noticed [123]. The alternation in gut microbiota and the bioavailability of anthoycanins by microbiota are two key elements in investigating the health benefits of anthocyanins. However, because collective knowledge of the gut microbiome still necessitates expansion, further research is needed to determine the association between the health benefits of anthocyanins and microbiota fermentation.

## 5. Conclusions

Epidemiology evidence, cell line investigations, and animal preclinical studies all showed the antitumor effects of anthocyanins in colon cancer. Anthocyanin-rich whole foods, anthocyanin extracts, anthocyanin single compounds, and the metabolites of anthocyanins all exhibited antitumor effects against CRC. The antitumor effects of anthocyanins are associated with multiple biological processes including proliferation, apoptosis, cell invasion, methylation, inflammation, and gut microbiota modification involving multiple genes and signaling pathways. The antitumor effects of anthocyanins are affected by their structures. Low bioavailability is a large pitfall in the research on anthocyanins. Phase I and phase II metabolites, conjugated products, and microbe-generated metabolites should be considered in the total bioavailability. The antitumor effects of anthocyanins may be attributed to the involvement of multiple genes, integrated roles in cell proliferation, apoptosis, invasion and metastasis, epigenetics, inflammation and gut microbiota modification. Using encapsulation strategies can increase the local bioavailability and target tissue specificity. Combining different anthocyanins, and anthocyanins with other polyphenols or drugs, and harnessing the gut microbiota fermentation, may enhance the health benefits of anthocyanins.

## Figures and Tables

**Table 1:** Anthocyanin-rich whole fruits and vegetables in colon cancer prevention.

Fruit	Model	Findings	Ref.
Lyophilized strawberries	AOM/DSS mouse	Decreased tumor incidence; reduced pro-inflammatory mediators and nitrosative stress; decreased PI3K/AKT signaling and NFkB	[16]
Lyophilized blackberriesand strawberries	AOM/DSS rat	Reduced the tumor number; reduced pro-inflammatory gut bacterial	[25]
Lyophilized acai pulp	1,2-dimethylhydrazine (DMH) and 2,4,6-trinitrobenzene acid (TNBS) rat	Reduces the total number of ACF, ACF multiplicity, tumor cell proliferation and incidence of tumors with high-grade dysplasia	[26]
BRB	AOM rat	Decreased ACF burden and aberrant crypt multiplicity; decreased adenocarcinoma multiplicity; reduced urinary 8-OHdG levels	[27]
Pomegranate juice	AOM rat	Suppressed the number of ACF and dysplastic ACF; lowered proliferation of mucosa cells; decreased expression of COX-2, iNOS, NF-κB (p65) and VCAM-1	[28]
Baked purple potato	AOM mouse	Suppressed tumor incidence; promoted apoptosis; increased cancer stem cell markers	[29]
Tart cherry	APC mouse	Decreased the number and volume of adenomas	[31]

**Table 2:** Whole fruit with anthocyanin components in colon cancer prevention.

Source	Model	Finds	Ref.
BRB	AOM/DSS mouse	Decreased tumor multiplicity; modulated the composition of gut commensal microbiota	[34]
BRB	HT-29, HCT116, Caco2, and SW480 cells	Inhibited cell growth; promoted apoptosis; suppressed demethylation effects	[32,33]
Blueberry	HT-29, HCT116, and Caco-2 cells	Inhibited cell growth; promoted apoptosis	[33,35,36]
*Vaccinium uliginosum* L.	DLD-1 and COLO205 cells	Inhibited cell proliferation	[37]
Bilberry	Caco-2, HT-29, and MC38 cells	Antioxidative; induced mitochondrial damage; activated cell apoptosis; inhibited cell growth; suppressed the activity of topoisomerase I; suppressed strand-breaking effects	[35,38,39,40,41,42]
Bilberry	AOM/DSS mouse	Decreased tumor number and inflammation	[43]
Bilberry extract	AOM rat	Reduced total ACF, colonic cellular proliferation, and COX-2 mRNA expression	[44]
Cranberry	HT-29, HCT116, SW480, and SW620 cells	Inhibited cell growth; promoted apoptosis	[33,45]
Chokeberry	HT-29 and Caco-2 cells	Inhibited cell growth; arrested G1/G0 and G2/M phases; increased expression of p21WAF1 and p27KIP1; decreased expression of cyclin A and cyclin B	[38,40,46,48,49]
Chokeberry	AOM rat	Reduced total ACF and colonic cellular proliferation	[44]
Blackberry	HT-29, HCT116, and CaCo-2 cells	Inhibited cell growth; promoted apoptosis; suppressed IL-12 release; suppressed peroxyl radical-induced cellular oxidative damage and apoptosis	[33,50,51]
Red raspberry and strawberry extract, lingonberry, elderberry, black currant	HT-29 and HCT116 cells	Inhibited cell growth; promoted apoptosis	[33,39,40]
Prunus spinosa drupes	HCT 116 cell, xenograft mouse	Inhibited growth and colony formation; promoted apoptosis in cells; reduced tumor growth in xenograft mice	[53]
Grapes	HT-29, CaCo-2, and HCT-116 cells	Inhibited cell growth; promoted apoptosis; suppressed the activity of topoisomerase I; suppressed strand-breaking effects; decreased expression of anti-apoptotic proteins survivin, cIAP-2, XIAP; arrested cells in G1; inhibited tyrosine kinase	[38,42,54,55,56]
Grape extract	AOM rat	Reduced total ACF, COX-2 mRNA expression	[44]
Red grape	APC^min^ mouse	Decreased adenoma burden and adenoma number; reduced the expression of AKT and Ki-67	[57]
Red wine	HCT-15 and HCT-116 cells	Suppressed cell growth; blocked S, G2, and M phase; reduced cell proliferation; increased p53 and p21	[58,59]
Tart cherry	APC—mouse	Decreased weight loss and tumor burden in combination with sulindac	[60]
Illawarra plum	HT 29 cell	Suppressed cell growth; arrested S cell phase; increased apoptosis; decreased telomerase activity and telomere length	[61]
Eugenia jambolana (Java plum)	HCT 116 cell	Suppressed cell proliferation; promoted apoptosis; elevated colon cancer stem cell	[62]
*Vitis coignetiae*	HT-29 and HCT-116 cells	Inhibited cell viability; induced apoptotic cells; inhibited cell growth; activated AMPKα1; inhibited mechanistic target of rapamycin (mTOR); inhibited invasive cells; suppressed NFkB-MMP-2/MMP-9 axis; inhibited cell migration, suppressed transepithelial electrical resistance	[63,64,65,66]
*Vitis coignetiae Pulliat*	Xenograft mouse	Inhibited tumor growth	[65]
Purple potato	Colon cancer stem cell	Suppressed proliferation; promoted apoptosis; suppressed WNT signaling pathway	[29]
Purple fleshed sweet potato	WiDr and SW480 cells	Inhibited cell growth; decreased cell number; arrested G1 phase	[67,68]
Sweet potato p40	AOM mouse	Decreased ACF number and cell proliferation; promoted apoptosis	[67]
Purple-shoot tea	HT-29 cell, COLO 320DM	Inhibited cell proliferation; arrested G0/G1 cell phase; promoted apoptosis; reduced cyclin E and cyclin D1; upregulated p21 and p27 cyclin-dependent kinase inhibitors; activated PAPR cleavage of caspase 3; increased Bax/Bcl-2 ratio	[69]
*Curly kale*	Caco-2, HT-29, and HCT 116 cells	Inhibited cell proliferation	[70]
*Pistacia atlantica sub kurdica*	HT 29 cell	Inhibited cell growth; arrested S phase	[72]
Chinese eggplant	HT 29 cell	Antioxidant, protected cell from DNA damage	[71]
Apple	Rat	Inhibited ACF, regulated apoptosis-related genes Aurka, p53 and COX-2, and cell migration-related genes MMP-2 and 9	[73]

**Table 3:** Anthocyanin or a single compound in colon cancer prevention.

Anthocyanins	Source	Model	Finds	Ref.
**Cyanidin**				
Cyanidin chloride Cyanidin chloride-3-5-diglucoside Cyanidin chloride-3 galactoside		HT29 cells	Inhibited the neurotensin- and EGF-induced increased rate of extracellular acidification; inhibited intracellular Ca^2+^ concentration induced by neurotensin	[75]
Cyanidin-3-*O*-b glucopyranoside			Inhibited cell growth and proliferation; increased ATM, topoisomerase II, HSP70 and p53 expression; increased DNA damage ROS-independent	[74]
Cyanidin		Caco-2,LoVo and LoVo/ADR (metastatic colon cancer cells)	Had cytotoxicity in metastatic cells, especially sensitive to drug-resistant LoVo/ADR	[76]
Cyanidin-3-glucoside	Strawberries	HT29 and HCT-116 cells	Anthocyanin extract had better antiproliferation effects on the cancer cells. Pure anthocyanin compounds differed in their efficacy on cell proliferation	[77]
Cyanidin-3-glucoside		HCT116, Caco2, and SW480 cells	Regulated the interaction of talin with β1A-integrin; promoted the attachment between colon cancer cells and fibronectin; inhibited 3D spheroid growth	[78]
Cyanidin-3-*O*-β-glucoside	Purple corn	DMH-PhIP rat	Decreased the development of colonic mucosal lesions and ACF induction	[79]
Cyanidin 3-cyanidin 2′′-*O*-β-D-glucopyranosyl-6′′-*O*-α-rhamnopyransyl-β-D—glucopyranoside 3-cyanidin 6′′-*O*-α-L-rhamnopyranosyl-β-D-glucopyranoside		APC—mouse HT-29 and HCT-116 cells	Decreased the number and volume of adenomas^–^; reduced cell growth	[31]
**Pelargonidin**				
Cyanidin-3-glucoside Pelargonidin Pelargonidin-3-glucoside	Strawberries	HT-29 and HCT-116 cells	Anthocyanin extract had better antiproliferation effects on cancer cells. Pure anthocyanin compounds differed in their efficacy cell proliferation	[77]
**Delphinidin**				
Delphinidin		CaCo-2 cell	Inhibited cell growth; arrested G1 phase	[80]
Delphinidin		HCT-116 cell	Decreased cell viability; promoted apoptosis; arrested G2/M phase; activated NFkB signaling	[81,82]
Cyanidin and delphinidin		Caco-2, LoVo and LoVo/ADR cells	Had cytotoxicity in metastatic cells, especially sensitive to drug-resistant LoVo/ADR	[76]
**Metabolites**				
Anthocyanin metabolites gallic acid, 3-*O*-methylgallic acid, syringic acid, protocatechuic acid, vanillic acid, and 2,4,6-trihydroxybenzaldehyde		Caco-2 cell	Anthocyanin metabolites gallic acid, 3-*O*-methylgallic acid, and 2,4,6 trihydroxybenzaldehyde reduce cell proliferation in Caco-2 cells more effectively than anthocyanins.	[83]
Malvidin-3-glucoside Anthocyains metabolites gallic acid, 3-*O*-methylgallic acid, and 2,4,6-trihydroxybenzaldehyde			Anthocyanin metabolites gallic acid and 3-*O*-methylgallic acid decreased cell viability; arrested G0/G1 phase; promoted apoptosis; caused DNA fragmentation and nuclear condensation	[84]
Anthocyanins digested in in-vitro GI model	Purple-fleshed sweet potato		Anthocyanin profile changed significantly after GI digestion	[85]
